# A comprehensive procedure for pollen extraction from bat guano deposits in organic and detrital matrices

**DOI:** 10.1016/j.mex.2023.102405

**Published:** 2023-10-02

**Authors:** Luiza Santos Reis, Paulo Eduardo de Oliveira, Qiang Yao

**Affiliations:** a University of São Paulo, Institute of Geoscience, Department of Sedimentary and Environmental Geology, São Paulo, Brazil; b The Field Museum of Natural History, Chicago, IL, United States; c Department of Oceanography and Coastal Sciences, College of the Coast and Environment, Louisiana State University, Baton Rouge, LA 70803, United States

**Keywords:** Pollen extraction from bat guano, Palynology, Bat guano, Pollen, Palynomorph, Paleoecology

## Abstract

Although bat guano deposits have been proven to be excellent environmental archives for paleoecological and paleoclimate studies, the development of a standardized method specially catering to pollen extraction has received no attention so far. In general, the processing procedure is quite similar among published studies, but adjustments must be made regarding the proportion of organic and particulate matter in the guano deposit. In this study, we present step-by-step optimized sample processing methods for pollen analysis. These procedures first apply a chemical treatment for the removal of siliceous and organic material, followed by a sieving step to remove the remaining inorganic matter from those samples with high detrital content. Overall, our methods can efficiently remove particulate matter and improve the quality of the final residue, resulting in cleaner slides and better visualization of pollen and spores.

• Remove humic acid and organic material with Potassium hydroxide.
• Remove inorganic matter with hydrofluoric acid and sieving.
• Concentrate and store the pollen residues in glycerin.

## Graphical abstract

**Figure:**
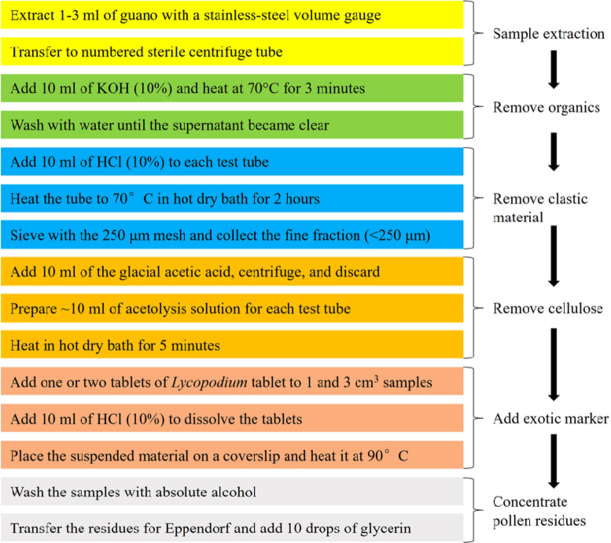

Specifications table

**Table:** 

Subject area:	Earth and Planetary Sciences
More specific subject area:	Palynology
Name of your method:	pollen extraction from bat guano
Name and reference of original method	N/A
Resource availability:	N/A

## Methods details

### Rationale

The extraction of pollen grains from sediment usually requires the use of a broad range of acids such as hydrofluoric acid (HF), hydrochloric acid (HCl), and sulfuric acid (H_2_SO_4_) to eliminate the mineral and organic content and separate the pollen and spores from their sedimentary matrix [1]. Regarding guano deposits, most existing protocols (Table 1) are effective in extracting pollen from organic-rich deposits [2]. In previous studies, pre-treatment with hot potassium hydroxide (KOH) is favored, which primarily facilitates the removal of organic matter. However, detritus-rich (e.g., iron concretions, insect shells, seeds, etc.) guano requires a different approach using a distinct chemical processing procedure, and such methods have rarely been mentioned in the literature.

**Table 1:** Comparison of pollen extraction protocols from existing literature.

Cave ID	Consulted Literature	Location	Samples	Sample volume	Chemical procedure
3	Maher, 2006	Tumbling Creek Cave, Missouri, USA	Bat guano core	Uninformed	(1) A wash with warm water and detergent;
					(2) Acetolysis.

7	Campbell et al., 2017	Fern Cave, Alabama, USA	Bat guano core	Uninformed	KOH (10%).

13	Carrión et al., 2006	southeastern Spain	Modern bat guano	2 g	(1) HCl;
					(2) HF;
					(3) KOH;
					(4) Mineral separation (ZnCl_2_)

15	Geantǎ et al., 2012	Măgurici Cave, NW Transylvania, Romania	Bat guano core	1 cm^3^	NaOH (30%)

15	Cleary et al., 2018	Măgurici Cave, NW Transylvania, Romania	Bat guano core	1 cm^3^	NaOH (30%)

16	Forray et al., 2015	Zidită Cave, western Romania	Bat guano core	2 cm^3^	NaOH (30%)

18	Cleary et al., 2019	Topolnița and Gura Ponicovei Caves, southwestern Romania	Bat guano core	1 cm^3^	(1) HCl;
					(2) NaOH (10%);
					(3) Sieving (250 μm).

21	Yang et al., 2021	southwestern China	Modern bat guano	5 g (dried sample)	(1) HCl;
					(2) Liquid flotation (KI/ZnI_2_);
					(3) Acetolysis.

22	Basumatary and Bera, 2014	Siju Cave of Meghalaya, India	Modern bat guano	Uninformed	(1) KOH (10%);
					(2) HF (40%);
					(3) Acetolysis.

**Fig. 1:**
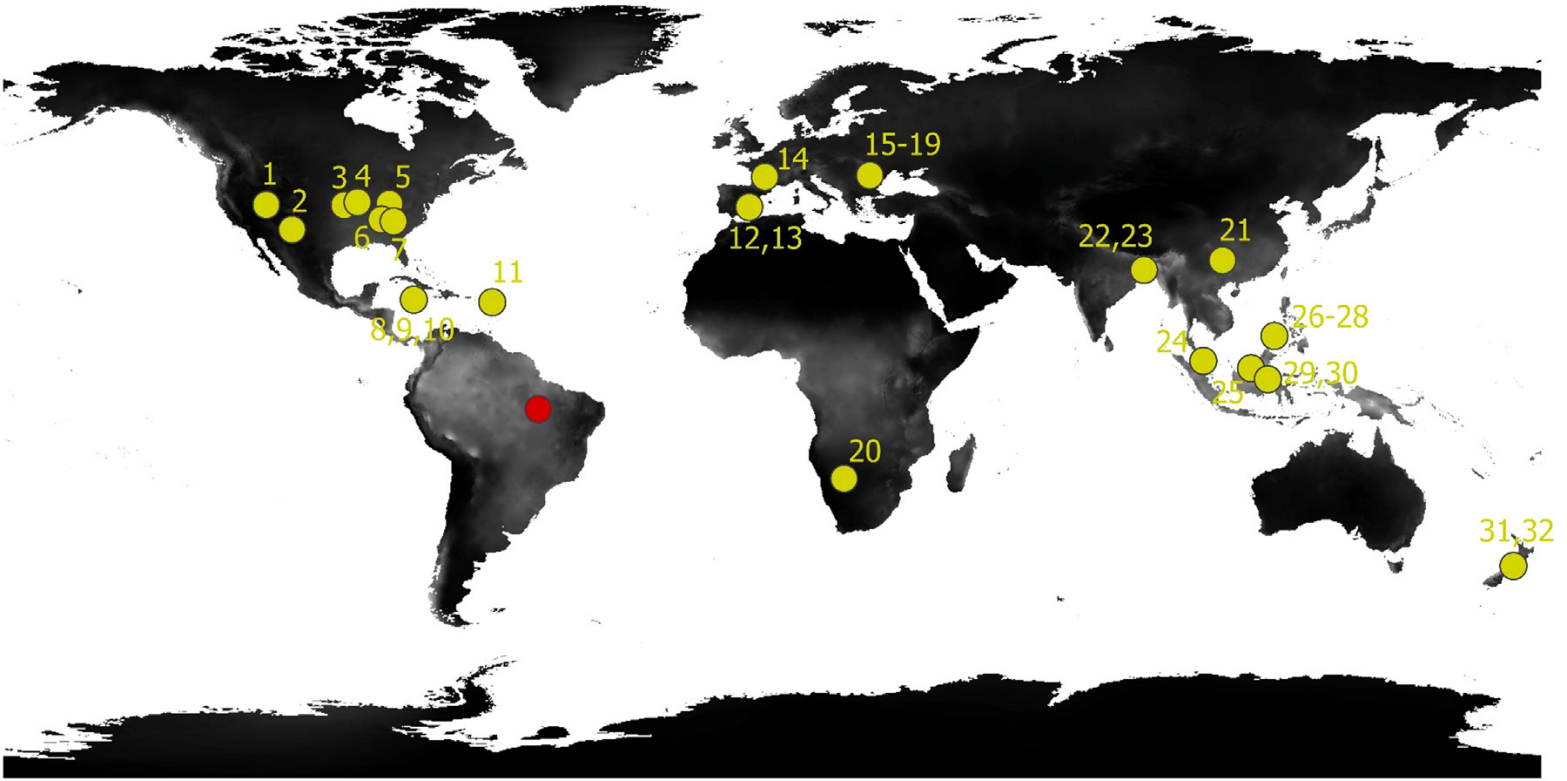
Locations of caves around the world where guano deposits have been studied. Red circle: our study site in Carajás, Brazil; Yellow circles: 1- Grand Canyon, Arizona, USA [5,6]; 2- Eagle Creek Cave, Arizona, USA [7,8]; 3- Tumbling Creek Cave, Missouri, USA [4]; 4- Round Spring Cavern, Missouri, USA [9]; 5- Mammoth Cave, Kentucky, USA [10]; 6- Spring Cave, Alabama, USA [11]; 7- Fern Cave, Alabama, USA [3]; 8- Jackson Bay Cave Complex, Jamaica [12,13]; 9- Schwallenburgh Cave, Jamaica [14]; 10- Home Away from Home Cave, Jamaica [14], [15], [16]; 11- Blanchard Cave, Eastern Caribbean [17]; 12,13- Southeastern Spain [18,19]; 14- Grotte XVI, France [20]; 15- Măgurici Cave, Romania [21], [22], [23]; 16- Ziditǎ Cave, Romania [24,25]; 17- Gaura cu Muscǎ Cave, Romania [26,27]; 18- Gura Ponicovei Cave, Romania [28]; 19- Topolnița Cave, Romania [29]; 20- Arnhem Cave, Namibia [30]; 21- China [31]; 22- Siju cave, India [32]; 23- Eraaning Cave, India [33]; 24- Batu Cave, Malaysia [34]; 25- Niah Cave, Borneo[34]; 26- Gangub Cave, Philippines [33]; 27- Makangit Cave, Philippines [34,35]; 28- Tabon Cave, Philippines [36]; 29- Gomantong Cave, Indonesia [37]; 30- Bau Cave, Indonesia [36]; 31- New Zealand [38]; 32- New Zealand [39].

Considering the growing number of studies on guano worldwide (Fig. 1), the lack of standardized pollen extraction protocols presents a major limitation to the development of cave palynology as it is evident that current processing methods (see Table 1) commonly employed to retrieve pollen and spores from bat guano cannot be applied to all types of guano deposit. Therefore, a specialized method is needed, especially for detritus-rich bat guano. Here, we present two different methods to retrieve pollen and spores from both inorganic and organic matrices. Our method for detritus-rich guano incorporates sieving as an extra key step comparing the standard chemical digestion pollen protocol [1], which markedly improves the preservation of pollen and spores from bat guano. While for purely organic guano, we present alternative steps to optimize the sample processing time and to obtain cleaner residues that will certainly make the pollen-counting process faster and easier.

### Geographic background of the sampling area

In this study, modern guano samples were retrieved from a bat cave in the plateaus of Serra Sul de Carajás (6°04″S, 50°10′W; Fig. 1), for both the experiment and control groups. The plateau towers over a forested terrain in the state of Pará, southeastern Amazonia. This region is part of the “Amazonian dry corridor”, characterized by its distinct monsoon climate that is divided into two clear wet and dry seasons [3]. Rainfall during the wet period, from November to May, ranges from 1545 mm to 1863 mm, while the dry period, from June to October, sees rainfall between 159 mm and 321 mm [3,4]. The average yearly temperature stands at 27.2 °C, dipping to 26.6 °C in January and peaking at 28.1 °C in September (Tavares et al., in 2018). The vegetation in the vicinity includes montane savanna, which encompasses open and woody terrains, as well as forested areas that are dominated by humid evergreen tropical forests, semi-deciduous woods, and isolated forest patches [4].

## Required materials and instruments

### General materials

**Fig. 2:**
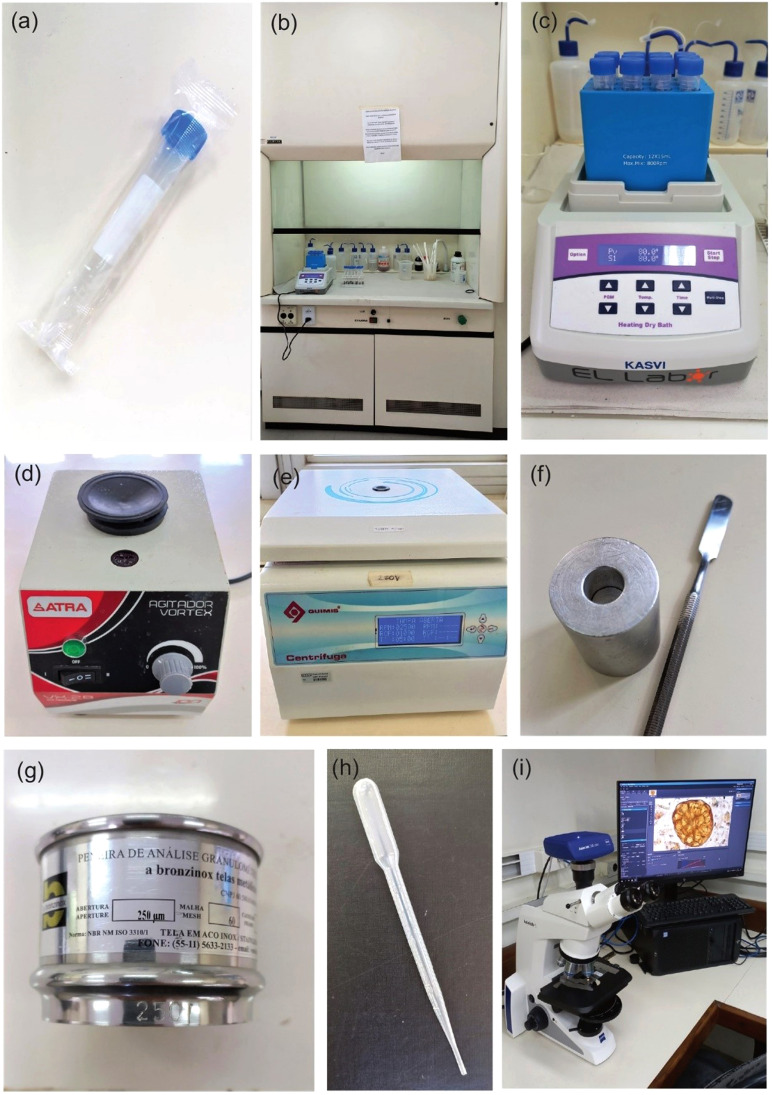
General materials and essential instruments for microfossil pollen preparation. (a) sterile centrifuge tube; (b) laboratory fume hood; (c) heating dry bath; (d) vortex mixer; (e) centrifuge; (f) 1 cm^3^ sampler and spatula; (g) 250 µm metal mesh sieve; (h) pasteur pipette 3 mL; (i) binocular light microscope.

1. Nitrile Exam or Vinyl Gloves

2. 15 mL sterile centrifuge tube (Fig. 2a)

3. 1 cm^3^ sampler and spatula (Fig. 2f)

4. Eye goggles

5. Respiratory mask

6. Measuring cylinder

7. Laboratory flasks

### Chemical materials

1. Deionized water

2. Hydrofluoric acid (HF)

3. Hydrochloric acid (HCl) (10 %)

4. Potassium hydroxide (KOH) (5 %)

5. Glacial acetic acid (CH_3_COOH)

6. Acetic anhydride (CH_3_CO)_2_O)

7. Sulfuric acid (H_2_SO_4_)

8. Absolute ethyl alcohol (C_2_H_6_O)

9. Glycerol (C_3_H_8_O_3_)

### Essential instruments

1. Acid-resistant fume hood (Fig. 2b)

2. Heating dry bath (Fig. 2c)

3. Vortex mixer (Fig. 2d)

4. Centrifuge (3500 RPM) (Fig. 2e)

5. 250 µm nylon or metal mesh sieve (Fig. 2g)

4. Pasteur pipette 3 mL (Fig. 2h)

5. Binocular light microscope (Fig. 2i)

## Procedures (all the steps should be performed inside a fume hood)

### Sampling

1. Sample the guano core at designated intervals. Scrap the surface clean or take material from inside the core where contamination is least likely. For surface samples, homogenize the samples before sampling. Pack samples into 1 cm^3^ sampler with a spatula, collect 1 cm^3^ for fully organic guano and 3 cm^3^ for guano with high detrital content, then transfer to numbered sterile centrifuge tube.

### Removal of humic acid and organic materials

2. Add 10 mL of KOH (10 %) to each tube, stir well, and heat in the hot (70 °C) dry bath for 3 min. KOH is used to deflocculate the sample, break down organic molecules, and remove humic compounds.

3. Centrifuge at 2500 rpm for 5 min, discard the supernatant and rinse with distilled water until the supernatant became clear. If the supernatant is still dark after three washes, step 2 and 3 should be repeated.

### Elimination of clastic materials with sieving and hydrofluoric acid

4. For guano with detrital content, add 10 mL of HF (hydrofluoric acid) to each test tube, stir well, and heat in the hot (70 °C) dry bath for 2 hr. Centrifuge, discard the supernatant, and rinse two times with distilled water. This step removes the clastic materials and dissolves silicates.

5. Sieve each sample with the 250 µm sieve and collect the fine fraction (<250 µm) of the sieved material. As most pollen grains are smaller than 250 µm in size, this step eliminates the clastic material with a diameter larger than 250 µm.

6. Centrifuge and discard the supernatant.

### Elimination of organic matter and cellulose

7. Add 10 mL of the glacial acetic acid, centrifuge, and discard the supernatant. This step acidifies the sediments and removes water, preparing the samples for acetolysis.

8. Prepare the acetolysis solution in a glass graduated cylinder. Use 9 parts acetic anhydride to 1 part concentrated sulfuric acid.

9. Add ∼10 mL of acetolysis solution to each test tube, stir well, and heat in the hot dry bath for 5 min. This step dissolves cellulose and removes organic matter. Centrifuge, discard the supernatant, and repeat step 7.

### Addition of exotic marker

10. Add one and two tablets of *Lycopodium clavatum* [40] to the 1 cm^3^ and 3 cm^3^ samples, respectively.

11. Add 10 mL of HCl (10 %) to dissolve the tablets. Centrifuge, discard the supernatant, and rinse two times with distilled water.

### Concentrate and store the pollen residues

**Fig. 3:**
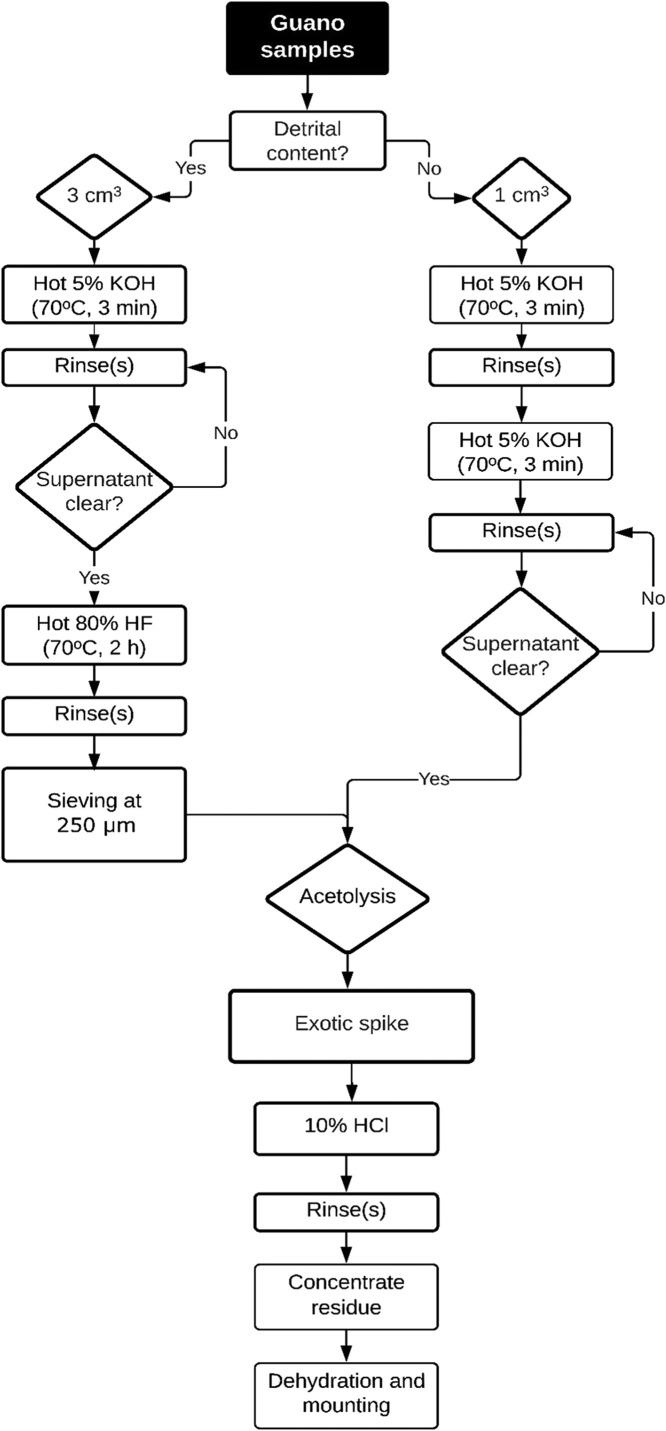
Flowchart of pollen preparation method used for guano samples and incorporating the sieving step.

12. Wash the samples with absolute alcohol, stir well, centrifuge, and discard the supernatant.

13. Transfer the residues for Eppendorf and add 10 drops of glycerin (C_3_H_8_O_3_).

14. Place the vials in a 50 °C oven and leave them uncovered overnight to allow the excess alcohol to evaporate.

15. On the following day, close the vials and store them in the refrigerator. The sequence of chemical treatments performed is outlined in Fig. 3.

## Method validation

**Fig. 4:**
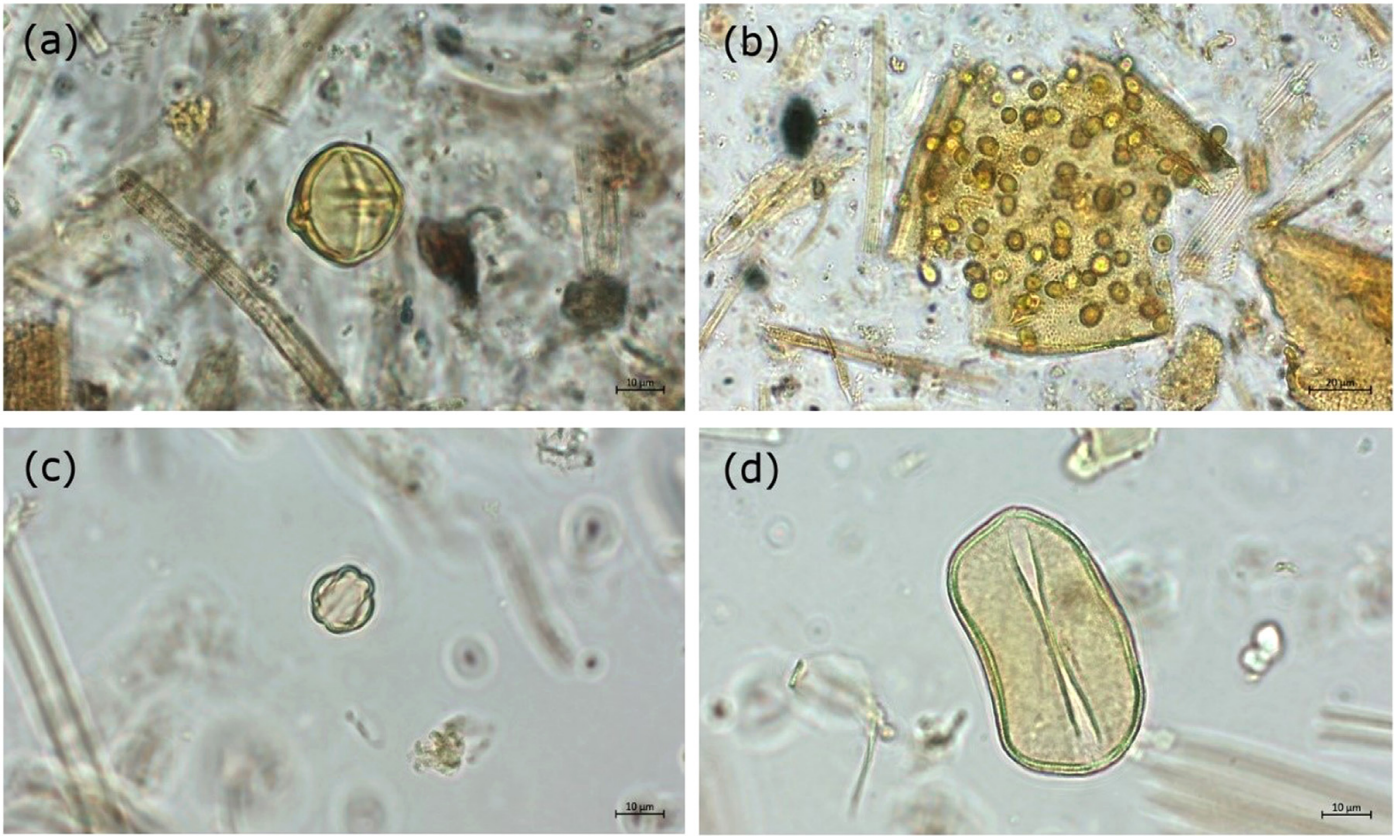
Micrographs of pollen grains at 63x and 100x magnification from bat guano with different preparation method. (a, b) samples without sieving; high proportion of minerals particles and organic matter. (c, d) this procedure after sieving and HF; lower proportion of organic and inorganic particulates. Pollen taxa: (a) *Solanum* sp.; (b) *Bauhinia* sp.; (c) *Miconia* sp.; (d) *Attalea* sp.

In a controlled experiment, we processed guano samples from the same bat cave but without the sieving procedure (Fig. 4a, b), and then we compared the results with samples processed using the procedure described in this study (Fig. 4c, d). The final residues and mounted slides demonstrate that our method, including chemical and sieving treatment, successfully separates large and small pollen grains from the matrix. For the control group, similar protocols without sieving yielded high organic and particulate matter in the final residue (Fig. 4a, b), which require additional steps to isolate pollen grains. In comparison, sieving the samples led to a much cleaner slide (Fig. 4c, d).

An additional advantage of including sieving is that pollen assemblages in the fine fraction are more concentrated and comprise ‘cleaner’ preparations than those from untreated samples, making the pollen counting process significantly faster and easier, hence, considerably reducing the amount of time an analyst needs to invest in pollen analysis. Similar results have been reported in the protocol for recovering *Zea mays* and other large-crop pollen after including an extra sieving treatment [41]. Moreover, our processing method for organic-rich samples do not require hazardous chemical reagents, such as hydrofluoric acid (HF), thereby less risky for the laboratory personnel when compared to the traditional acid-based procedure.

### Perspectives for a better pollen recovery

According to Whitney et al. (2012), the most important factors in ensuring a good recovery of pollen grains are (1) choosing the appropriate chemical treatments for each material type and composition, and (2) sufficient rinsing with distilled water to remove chemical residues after each treatment. Although our method works well for fully organic samples, demonstrated by a series of bat guano pollen-rain records from across the southeastern Amazonia [42], we encourage the insertion of a sieving step (250 µm) as proposed by Cleary et al. (2019) to further remove coarse organic matter and minerals, as well as facilitate subsequent chemical treatments and improve the quality of the final residue.

**Fig. 5:**
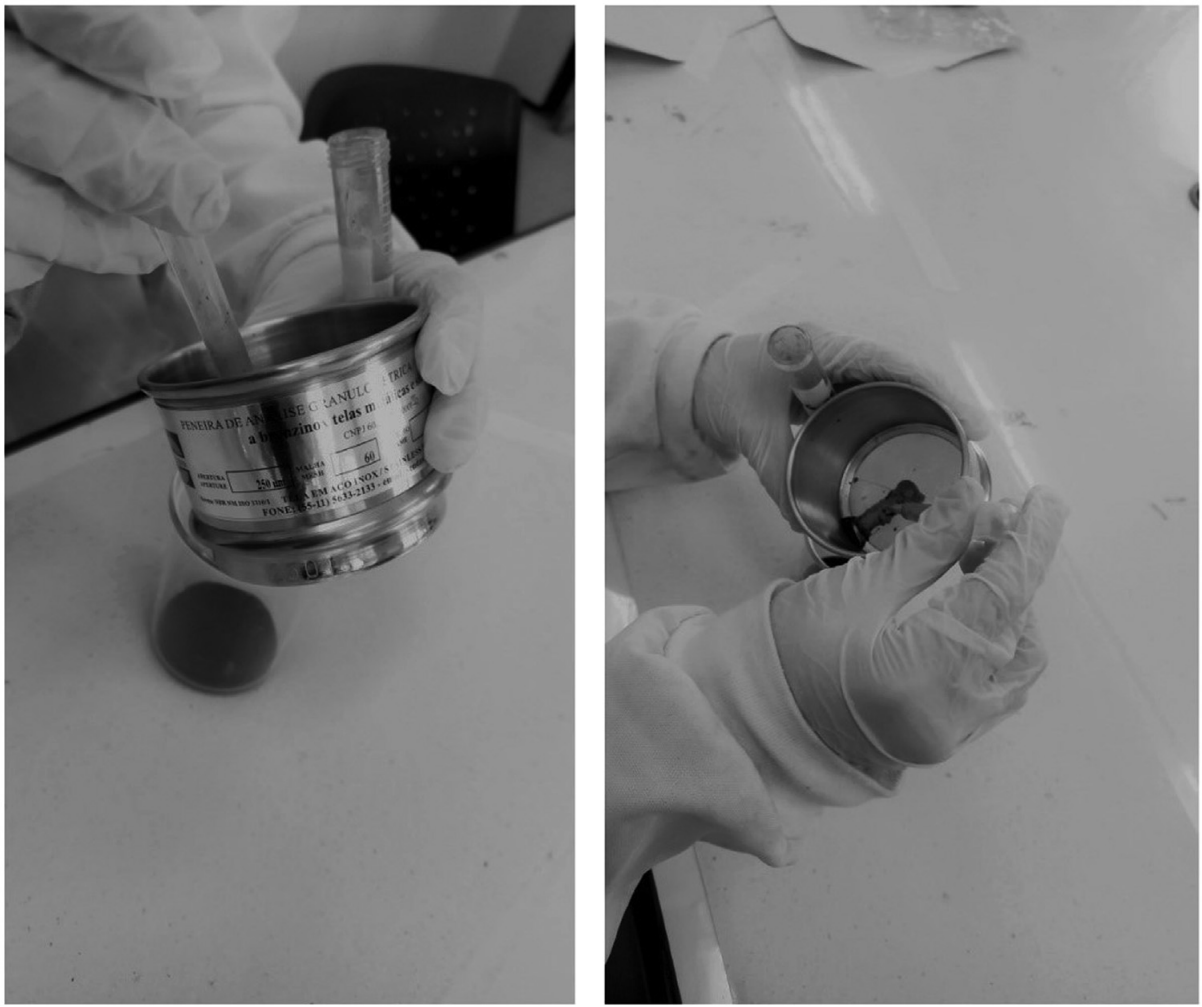
Sieving step using pipettes to speed up filtration and reduce the final water volume.

We also advise against using large volumes of water during the sieving step, which increases the number of centrifugation rounds and consequently prolong processing time. Alternatively, we recommend using pipettes to speed up the separation (Fig. 5) and a good wash of the tubes may result in a volume of filtrate that can be easily concentrated in 15 mL tubes, thus reducing the duration of sample processing.

### Implications for paleoecological and paleoenvironmental studies in Amazonia

In the pollen literature, most palaeoecological studies rely on terrestrial pollen and charcoal records. Where there is a paucity of pollen-based paleoenvironmental reconstruction from lake, wetland, and swamp habitats, bat guano deposits provide an additional archive that fill this data for paleoenvironment reconstruction and paleoecology studies. Over millennial timescale, several meters of guano can accumulate on cave floors [43], representing an outstanding environmental archive that becomes increasingly attractive for palynologist worldwide [5,^6^,^10^,^35^,44].

A growing number of studies on bat guano deposits is leading to successful environmental reconstructions [5,6], as well as unique information about archeological sites [3] and shifts in bat diets [45]. Paleoecological data from guano deposits have been reported by previous studies in North America [9,10] and Asia [32,35]. They provide valuable records of climate changes in the geological past [5], and bat ecology [4], among other environmental parameters throughout the last several millennium [6,35]. For example, geochemical and pollen data provided by a guano deposit from Romania revealed shifts in food supply and bats vacating the cave associated with changes in climate conditions during the Medieval Climate Anomaly and Little Ice Age [21,28]. However, paleoecological records are absent from bat guano deposits in Brazilian caves, which prevents further understanding of the couple bat-plant-climate interaction in biodiversity hotspots across the Globe South [42,46].

Among many research topics, this improved technique for recovering fossil pollen, and eventually modern pollen and spores, from guano will allow palaeoecologists to maximize their chances of assessing bat diets and the effects of past climate and environmental changes on the Neotropical bat communities.

## Conclusions

Our study shows that fossil pollen grains can be routinely extracted from high detrital-content guano by combining chemical pre-treatment and sieving. In organic-rich guano, the quality of extracted pollen strongly supports the effectiveness of our method as an alternative to the traditional acid-based procedures. Therefore, non-acid preparation techniques would be preferable, as they are less hazardous to laboratory personnel and more environmentally friendly. Additionally, more investigations should be undertaken using sieves with a mesh size of 125 µm to further prove its feasibility in palynological processing. Because modern and fossil pollen grains from bat guano can provide outstanding information regarding bat-plant interactions over time, these new methods will certainly contribute to future studies in paleoclimatology, paleoecology, and paleoenvironmental reconstructions.

## Ethics statements

This study does not involve any human subjects or animal experiments.

## CRediT authorship contribution statement

**Luiza Santos Reis:** Methodology, Writing – original draft. **Paulo Eduardo de Oliveira:** Supervision. **Qiang Yao:** Writing – review & editing.

## Declaration of Competing Interest

The authors declare that they have no known competing financial interests or personal relationships that could have appeared to influence the work reported in this paper.

## Data Availability

No data was used for the research described in the article.
